# Genetic background is associated with distinct patterns of proarrhythmogenic remodeling leading to atrial fibrillation in pigs with ischemic heart failure

**DOI:** 10.1007/s00395-026-01176-7

**Published:** 2026-04-08

**Authors:** Zhihao Zhang, Julia Vlcek, Christina Heger, Valerie Pauly, Nora Hesse, Dominik Schüttler, Daphne Merkus, Eckhard Wolf, Ivica Medugorac, Stefan Kääb, Philipp Tomsits, Sebastian Clauss

**Affiliations:** 1 Department of Medicine I, University Hospital, LMU Munich, Munich, Germany; 2 Interfaculty Center for Endocrine and Cardiovascular Disease Network Modelling and Clinical Transfer (ICONLMU), LMU Munich, Munich, Germany; 3 Institute of Surgical Research at the Walter-Brendel-Centre of Experimental Medicine, University Hospital, LMU Munich, Munich, Germany; 4 DZHK (German Centre for Cardiovascular Research), Partner Site Munich, Munich Heart Alliance (MHA), Munich, Germany; 5 Molecular Animal Breeding and Biotechnology, Gene Center and Department of Veterinary Sciences, LMU Munich, Munich, Germany; 6 Center for Innovative Medical Models (CiMM), LMU Munich, Oberschleißheim, Germany; 7 Population Genomics Group, Department of Veterinary Sciences, LMU Munich, 82152 Martinsried, Germany; 8 Department of Cardiology, Erasmus MC, Rotterdam, The Netherlands; 9 Department of Cardiology, First Affiliated Hospital of Zhengzhou University, Zhengzhou, Henan China

**Keywords:** Atrial fibrillation, Proarrhythmic remodeling, Fibrosis, Ischemic heart failure, Genetics, Pig model

## Abstract

Atrial fibrillation (AF) is the most common sustained cardiac arrhythmia, driven by electrical and structural remodeling that promotes proarrhythmic substrates. Interindividual variability in susceptibility to remodeling triggers, such as myocardial ischemia, suggests the involvement of genetic determinants. Ischemic heart failure (IHF) was induced by a 90-min occlusion of the left anterior descending artery in pigs. After 30 days, animals underwent in vivo assessments, including right and left heart catheterization, electrocardiography, and electrophysiological studies, with AF inducibility tested by burst pacing. Atrial fibrosis was quantified using Masson’s trichrome staining, and remodeling-associated gene expression was analyzed by quantitative PCR. Genetic background was assessed through single-nucleotide polymorphism (SNP) genotyping using the Porcine SNP60 BeadChip, with data analyzed against reference SNP datasets. Following myocardial infarction, all animals developed IHF with reduced ejection fraction and increased AF susceptibility. Overall, IHF was associated with enhanced atrial fibrosis, but a subset of animals displayed no fibrotic remodeling. Genetic analysis identified two groups of pigs with different genetic backgrounds within the cohort: a Pietrain-dominant and a Landrace-dominant background. Pietrain-dominant pigs developed significantly more atrial fibrosis, whereas Landrace-dominant pigs exhibited a pronounced shortening of the atrial effective refractory period. Differential regulation of fibrosis-associated genes, including *FN*, *MMP2*, *TGFB*, and *JNK1*, was observed between genetic backgrounds. These findings indicate that genetic background is associated with distinct patterns of atrial remodeling in response to IHF, influencing key determinants of AF susceptibility.

## Introduction

Atrial fibrillation (AF) is the most common sustained arrhythmia, affecting millions of people worldwide, with its prevalence projected to double in the coming decades [56]. AF is associated with substantial morbidity, including a fivefold increased risk of stroke, a twofold higher risk of dementia, and a threefold elevated risk of heart failure [42]. Moreover, AF doubles the risk of all-cause mortality [1]. Given its rising prevalence and high morbidity, AF imposes a significant burden on both individuals and healthcare systems [15]. The primary pathophysiological hallmarks of AF include enhanced ectopic activity and electrical reentry [58]. The former typically results from increased automaticity or dysregulated calcium homeostasis, while the latter is often driven by atrial fibrosis, which creates conduction barriers and alters refractoriness [34]. These mechanisms are summarized as electrical and structural remodeling and establish a proarrhythmic substrate for AF [12]. Major risk factors for AF include obesity, metabolic syndrome, obstructive sleep apnea, and cardiovascular diseases such as heart failure [7, 56], with myocardial ischemia being the most common cause of heart failure and an important determinant of AF incidence and prognosis [6].

Despite similar exposure to common risk factors, individual susceptibility to AF varies substantially [64]. This observation supports the ‘two-hit’ hypothesis, suggesting that a genetic predisposition modulates AF risk in the presence of additional environmental or clinical stressors [13].

In line with this concept, our previous work in pigs with ischemia-induced AF [11] demonstrated that regional heterogeneity of atrial fibrosis correlated with AF inducibility [65]. However, AF also occurred in a subset of animals with minimal fibrotic remodeling and without pronounced regional differences, suggesting that additional mechanisms—potentially associated with genetic background—contribute to AF pathogenesis.

Consistent with this notion, genome-wide association studies (GWAS) have identified numerous genetic loci linked to ion channel function, intracellular calcium regulation, and extracellular matrix remodeling, underscoring a substantial genetic contribution to AF [30, 35]. Since the initial identification of a common risk variant near PITX2 on chromosome 4q25 by Gudbjartsson et al. increased AF risk by up to 1.72-fold per allele, affecting 35% of a European cohort and 75% of a Chinese cohort [21], potentially through alterations in action potential dynamics [52], more than 350 risk loci associated with AF susceptibility have been reported [26, 44]. Multi-ethnic studies have further demonstrated that AF risk is significantly lower in non-white populations, including Black, Hispanic, and Asian individuals, compared to White populations [14], even after adjusting for a comparable burden of risk factors [53]. This genetic diversity contributing to AF susceptibility suggests substantial heterogeneity in its underlying pathophysiology, which likely contributes to variable responses to antiarrhythmic drugs and catheter ablation therapy [38]. Thus, elucidating the genetic mechanisms driving AF is essential for enabling mechanism-based treatment strategies and developing personalized therapeutic approaches with improved efficacy [38]. Although large GWAS have identified more than 350 genetic loci associated with AF [35, 44], the underlying pathophysiological mechanisms linking genetic variation to AF development remain incompletely understood.

Large prospective cohort studies, including analyses from the UK Biobank, have recently integrated genetic predisposition with acquired risk factors, such as diabetes, hypertension, and myocardial infarction, providing strong evidence for gene–environment interactions in AF susceptibility [60, 63]. Nevertheless, mechanistic preclinical studies addressing how genetic variation modulates AF substrates remain scarce [38]. Experimental animal models offer a controlled platform to address this gap, yet most studies have relied on genetically homogeneous models and have focused on either electrical or structural remodeling in isolation. Recent large-animal studies indicate that the genetic background of pigs critically influences cardiac responses to ischemia/reperfusion independent of overt comorbidities. Lean Ossabaw minipigs exhibit a genetically determined non-responder phenotype to ischemic preconditioning, characterized by absent infarct size reduction and failure to activate canonical cardioprotective pathways, including STAT3 signaling [27, 29]. In parallel, genetically driven alterations in vascular reactivity—manifesting as enhanced vasoconstriction and impaired endothelium-dependent and -independent vasodilation—have been described even before the development of metabolic disease, suggesting an inherent susceptibility to microvascular dysfunction during reperfusion [17, 28]. Together, these findings demonstrate that inherited traits shape myocardial and vascular phenotypes in response to ischemia/reperfusion in pigs. However, whether genetic background similarly determines atrial remodeling patterns and arrhythmogenic substrates following ischemic heart failure remains unresolved. To address this question, we utilized our well-established porcine model of ischemic heart failure (IHF) and AF [3, 11, 41, 47, 65] to investigate the impact of genetic background on ischemia-induced atrial remodeling and the resulting electrophysiological phenotype.

## Materials and methods

### Animals

All animal experiments were performed following the “Guide for the Care and Use of Laboratory Animals” and were approved by the *Regierung von Oberbayern*. 40 pigs aged 3–4 months were obtained from the *Landwirtschaftliche Forschungsstation Thalhausen* [Technical University of Munich (TUM), Kranzberg, Germany], the Moorversuchsgut, the Center for Innovative Medical Models [(CiMM), Ludwig-Maximilians-University (LMU), Oberschleissheim, Germany], and the Lehr- und Versuchsgut of the LMU (Oberschleissheim, Germany). To minimize the number of experimental animals (3R concept), the 40 pigs used in this study have already been used for a previous publication [65] but the data presented in the current study result from an entirely new genotype-specific analysis.

### Pig model

The experimental protocol used to induce IHF has been described in detail previously [11, 47]. Briefly, pigs were sedated with an intramuscular injection of ketamine (20 mg/kg), azaperone (10 mg/kg), and atropine (0.05 mg/kg). Once sedation was achieved, intravenous access was established in the outer auricular vein, followed by the administration of midazolam (0.5 mg/kg) to induce anesthesia, and propofol (0.5 mg/kg/min) along with fentanyl (0.5 mg/kg) to maintain anesthesia. After preparing the surgical site with the pig in dorsal recumbency, 8F and 9F sheaths were inserted into the right external jugular vein and right carotid artery. The left anterior descending coronary artery was then occluded using an angioplasty balloon distal of the first diagonal branch for 90 min. The location of the occlusion was confirmed by X-ray. After the reperfusion period, all catheters and sheaths were removed, and the surgical site was carefully closed. Animals were then transferred to the housing facility and monitored for 30 days.

### Invasive hemodynamic assessment

To assess ejection fraction, levocardiography was performed using a 6F pigtail catheter in the left anterior–oblique (LAO) view at 30° and the anterior–posterior view at 0°. Left-ventricular pressure and end-diastolic pressure were also measured invasively. Pulmonary artery pressure, pulmonary capillary wedge pressure, right atrial pressure, and right-ventricular pressure were assessed using a Swan–Ganz catheter through a venous sheath. All assessments were conducted at a paced heart rate of 130 bpm with 1:1 atrioventricular conduction.

### Electrocardiography and electrophysiological studies

Heart rate was monitored using 12-lead electrocardiography (ECG). Electrophysiological studies were conducted with the MLCL CardioLab System (GE Healthcare, USA). A multipolar electrophysiology catheter was placed in the high right atrium to perform atrial stimulation at 2× pacing threshold. Sinus node recovery time (SNRT) was measured as the interval between the last pacing stimulus and the first intrinsic atrial signal. To assess atrioventricular conduction properties, the Wenckebach cycle length (WCL), atrioventricular effective refractory period (AVERP), and atrial effective refractory period (AERP) were measured. The Wenckebach point was determined by progressively shortening the pacing cycle length until a 1:1 atrioventricular conduction could no longer be maintained. AERP and AVERP were measured using a series of seven fixed stimulations followed by an eighth single premature pacing stimulus with stepwise shortening of the pacing cycle length. AERP was defined as the pacing cycle length that failed to induce a P wave, while AVERP was defined as the cycle length that failed to conduct to the ventricle. Finally, arrhythmias were induced by a series of rapid burst pacings at 1200 bpm for 6 s. AF was defined as an arrhythmic episode with irregular RR intervals lasting longer than 10 s.

### Histological assessment of structural remodeling

Structural remodeling was assessed by quantifying interstitial fibrosis in the left and right atria, as well as in the ventricles (remote from the infarcted area). Tissue samples were prepared as 5 µm paraffin-embedded sections, followed by Masson’s Trichrome staining using the Masson–Goldner Trichrome staining kit (Carl Roth GmbH + Co. KG, Germany). Ten non-overlapping images per sample were acquired with a high-resolution microscope (DM6 B, Leica, Germany) using a 40-fold objective. Interstitial fibrosis was then analyzed by a blinded observer.

### Genotyping

Genetic background was assessed through genotyping of single-nucleotide polymorphisms (SNPs) using the PorcineSNP60 BeadChip (Illumina). The dataset containing 46,298 informative SNPs was divided into blocks of 4 SNPs and used to estimate the allele-sharing distances between 40 pigs in this study and purebred reference breeds (German Landrace and Pietrain) as described previously [39]. The 4-SNP blocks (haplotypes) were defined to span less than 150 kilobases and to have a distance between markers of less than 50 kilobases, resulting in a compromise between the maximum number of SNPs and the minimum recombination probability within the block. The matrix of allele-sharing distances was projected onto the plane using multidimensional scaling (MDS) and used to determine the genetic background of 40 pigs examined here in relation to purebred references in the existing SNP database. Pairwise distances between individuals were visualized using a clustered heatmap in R (R Core Team, 2024). Heatmaps were generated using the *ComplexHeatmap* package [20], and phylogenetic representations of hierarchical clustering (UPGMA) were handled using the ape package [40]. Genetic background assignment determined by MDS and complex heat maps was not used to guide experimental allocation but served exclusively for post hoc stratified analyses of remodeling phenotypes.

### Quantitative polymerase chain reaction (qPCR)

Total RNA isolation was carried out using TRIzol reagent (Invitrogen, Life Technologies, USA) according to the manufacturer’s standard protocol. The obtained RNA was quantified spectrophotometrically using a NanoDrop, and samples were diluted to a concentration of 50 ng/µl for cDNA synthesis. cDNA samples were prepared using the SuperScript IV Reverse Transcriptase Kit (Thermo Fisher Scientific, USA), following the manufacturer’s instructions. qPCR was performed by iTaq Universal SYBR Green Supermix (Bio-Rad, USA), according to the manufacturer’s protocol. All qPCR primers are listed in Table 1. The Ct values were normalized to the Ct value of *ACTB*, and gene expression levels were quantified as relative fold changes using the 2^ΔΔCT^ method [62].

**Table 1:** qPCR primer sequences

Gene	Primer sequences
*ACTB*	Forward: TCTGGCACCACACCTTCT
	Reverse: TGATCTGGGTCATCTTCTCAC
*ACTA2*	Forward: TGAGCTTCGTGTTGCCCCA
	Reverse: CAGCACCGCCTGAATAGCCA
*CCN2*	Forward: GTCAGGCCTTGTGAAGCTGA
	Reverse: GCCCGGTATGTCTTCACACT
*cJUN*	Forward: CAAGGCGGAGAGGAAGCGTA
	Reverse: GTTCCCTGAGCATGTTGGCG
*COL1A1*	Forward: ACCTCAAGATGTGCCACTCC
	Reverse: CCTGTCTCCATGTTGCAGAA
*FN*	Forward: TTCATGTCATCCCGTGGGCA
	Reverse: GACCCGTCAAGGTGGCACTA
*FSP1*	Forward: GGTGTGACGCTGGTATTTGTTTGAA
	Reverse: AGGGAGGAGAAAGCGGATGAAC
*MMP2*	Forward: CTTTGATGGCAAGGACGGGC
	Reverse: TCACACGCACCACTTGTCCT
*JNK1*	Forward: ACCTGACAAGCAGTTGGATGA
	Reverse: CCTGTGCTAAAGGAGAGGGC
*TGFB*	Forward: CTGGAAAGCGGCAACCAAAT
	Reverse: GCTCTGCCCGAGAGAGCAATA

### Statistical analysis

Data are presented as mean ± SEM. Statistical analysis was performed using GraphPad Prism 9. A non-parametric Mann–Whitney *U* test was conducted to determine differences between two groups. Fisher’s exact test was used to compare categorical variables. Results were considered statistically significant with a *p* value of < 0.05. Given the exploratory and hypothesis-generating nature of the study, no formal correction for multiple comparisons was applied; statistical analyses were interpreted descriptively with appropriate caution.

## Results

### Differences in fibrotic remodeling and AF substrates by genetic background in IHF pigs

**Fig. 1:**
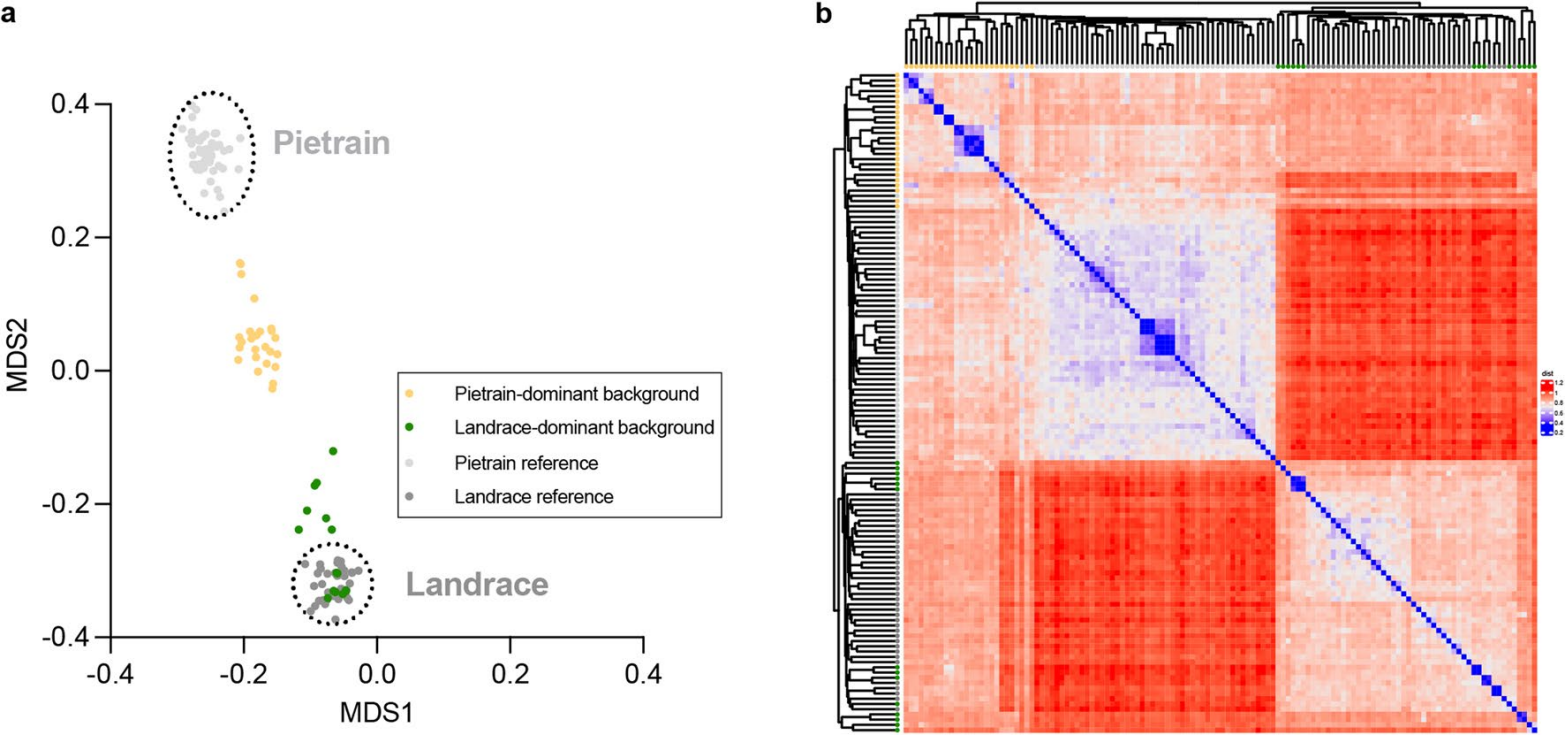
The classification of pigs was performed based on their genetic background, dividing them into two groups: those with a Pietrain-dominant background and those with a Landrace-dominant background. **a** The genome-wide allele-sharing distance matrix between all animals, scaled down to the two-dimensional plane by multidimensional scaling (MDS). **b** The complex heatmap comprises 127 animals (49 purebred Pietrain and 38 German Landrace and 40 experimental pigs). Higher genetic relationship is depicted in blue (i.e., low genetic distance), whereas high genetic distance (low relationship) is shown in red. A hierarchical clustering dendrogram (UPGMA) is displayed on both axes to illustrate phylogenetic relationships between individuals

**Fig. 2:**
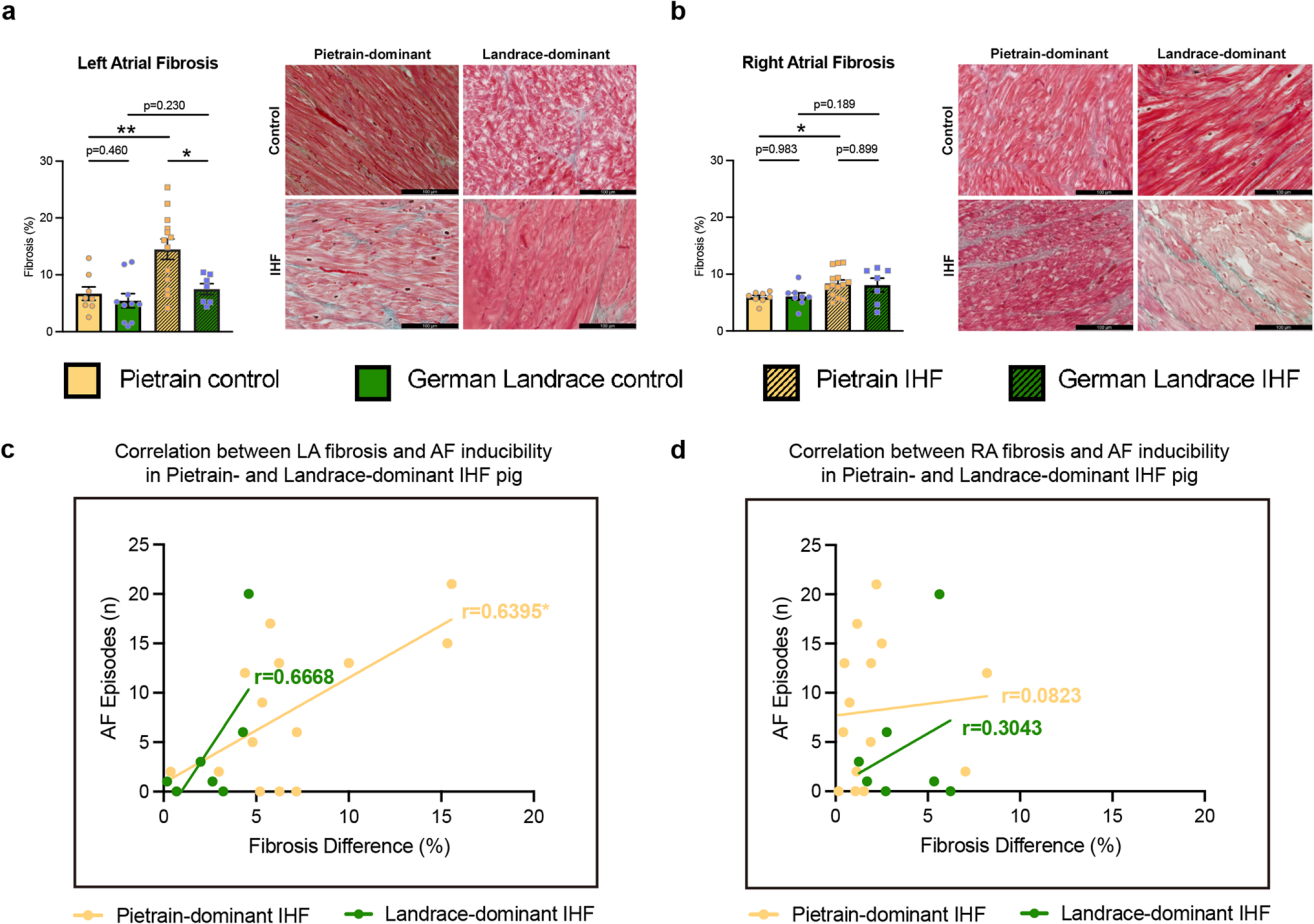
Differential fibrotic remodeling and AF substrates with different genetic backgrounds in IHF pigs. Quantification of fibrosis and representative images in the **a** left and **b** right atria from Pietrain- and Landrace-dominant control and IHF pigs, scale bar 100 µm; correlation of the number of AF episodes with **c** left and **d** right atrial fibrosis difference in Pietrain- and Landrace-dominant IHF pigs. Bar graphs represent mean ± SEM, and each circle represents individual pig data. Group comparison was performed using Mann–Whitney *U* test. *p < 0.05, **p < 0.01. Pearson correlation analysis was applied for evaluating associations. *p < 0.05

In our previous study, pigs developed significant IHF and showed an increased susceptibility to AF 30 days after myocardial infarction [65]. Histological analysis revealed a significant increase in both left and right atrial fibrosis in IHF pigs compared to controls [65]. Furthermore, we observed that not the absolute levels of fibrosis, but regional heterogeneity of fibrosis distribution correlated with AF burden [65]. However, in some pigs, no increase in atrial fibrosis or fibrosis heterogeneity was observed, although these pigs showed a similar IHF and AF phenotype. Given that previous work suggested the genetic background affecting ischemia-mediated remodeling [50], we assessed the genetic background of the pigs that were studied. Analyses of genome-wide allele-sharing distances using multidimensional scaling and complex heat maps reveal clear patterns in the 40 pigs examined here. Including whole-genome SNP genotypes of purebred German Landrace and Pietrain pigs as a reference helped us to identify two groups of pigs in our material, one with a predominantly Pietrain genetic background and the other with a predominantly German Landrace genetic background (Fig. 1). Re-analyzing our data and taking the genetic background into account demonstrated that Pietrain-dominant IHF pigs exhibited a significant increase in atrial fibrosis, whereas this was absent in Landrace-dominant IHF pigs. Moreover, in Pietrain-dominant IHF pigs, LA fibrosis heterogeneity was significantly correlated with the AF phenotype, which was not observed in Landrace-dominant IHF pigs (Fig. 2).

### The ischemic heart failure phenotype following LAD occlusion is similar in both Pietrain-dominant and Landrace-dominant pigs

**Fig. 3:**
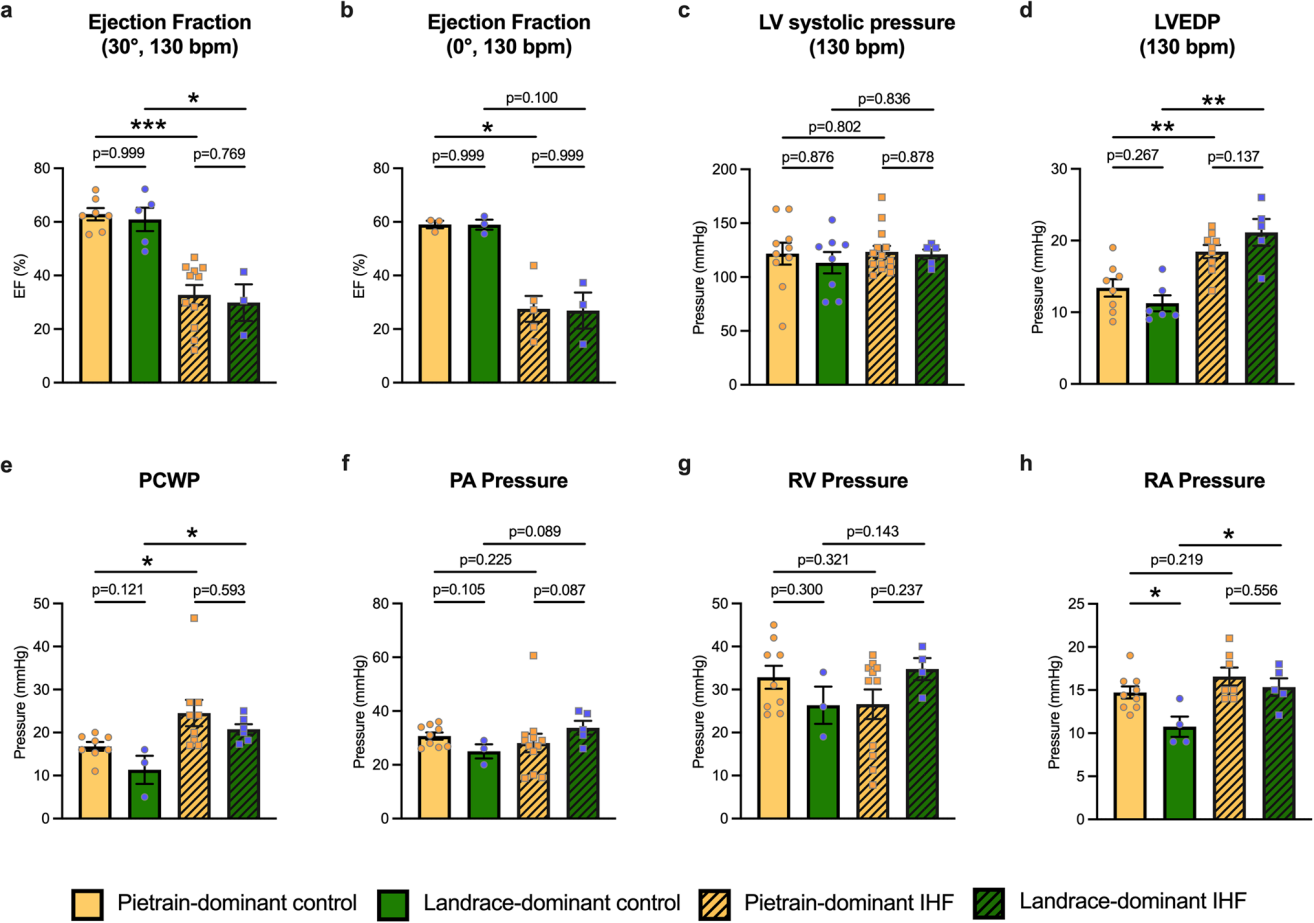
Myocardial infarction induced significant ischemic heart failure (IHF) in both Pietrain- and Landrace-dominant pigs as confirmed by hemodynamic measurements. **a** Ejection fraction measured by levocardiography at 130 bpm in the left anterior oblique (LAO) 30° and **b** anterior–posterior (AP) 0° views; **c** left-ventricular (LV) systolic pressure; **d** left-ventricular end-diastolic pressure (LVEDP); **e** pulmonary capillary wedge pressure (PCWP); **f** pulmonary artery (PA) systolic pressure; **g** right-ventricular (RV) systolic pressure; **h** right atrial (RA) systolic pressure. Bar graphs represent mean ± SEM, and each circle represents individual pig data. Mann–Whitney *U* test. *p < 0.05, **p < 0.01, ***p < 0.001

To assess whether the genetic background affects the development of IHF, cardiac function and hemodynamic parameters were evaluated in both Pietrain- and Landrace-dominant animals. Both groups exhibited a similar IHF phenotype demonstrated by a significantly reduced ejection fraction (EF) (Fig. 3a and b). Correspondingly, both IHF pig groups showed elevated left-ventricular end-diastolic pressure (LVEDP) compared to their respective controls (Fig. 3d), as well as increased pulmonary capillary wedge pressure (PCWP) (Fig. 3e). In addition, Landrace-dominant IHF pigs showed significantly increased right atrial pressure, while Pietrain-dominant IHF pigs had no difference compared to control. There were no significant differences in left-ventricular systolic pressure, pulmonary artery pressure, and right-ventricular pressure between the groups (Fig. 3c, f–h).

### Atrial fibrillation inducibility in Pietrain-dominant and Landrace-dominant IHF pigs

**Fig. 4:**
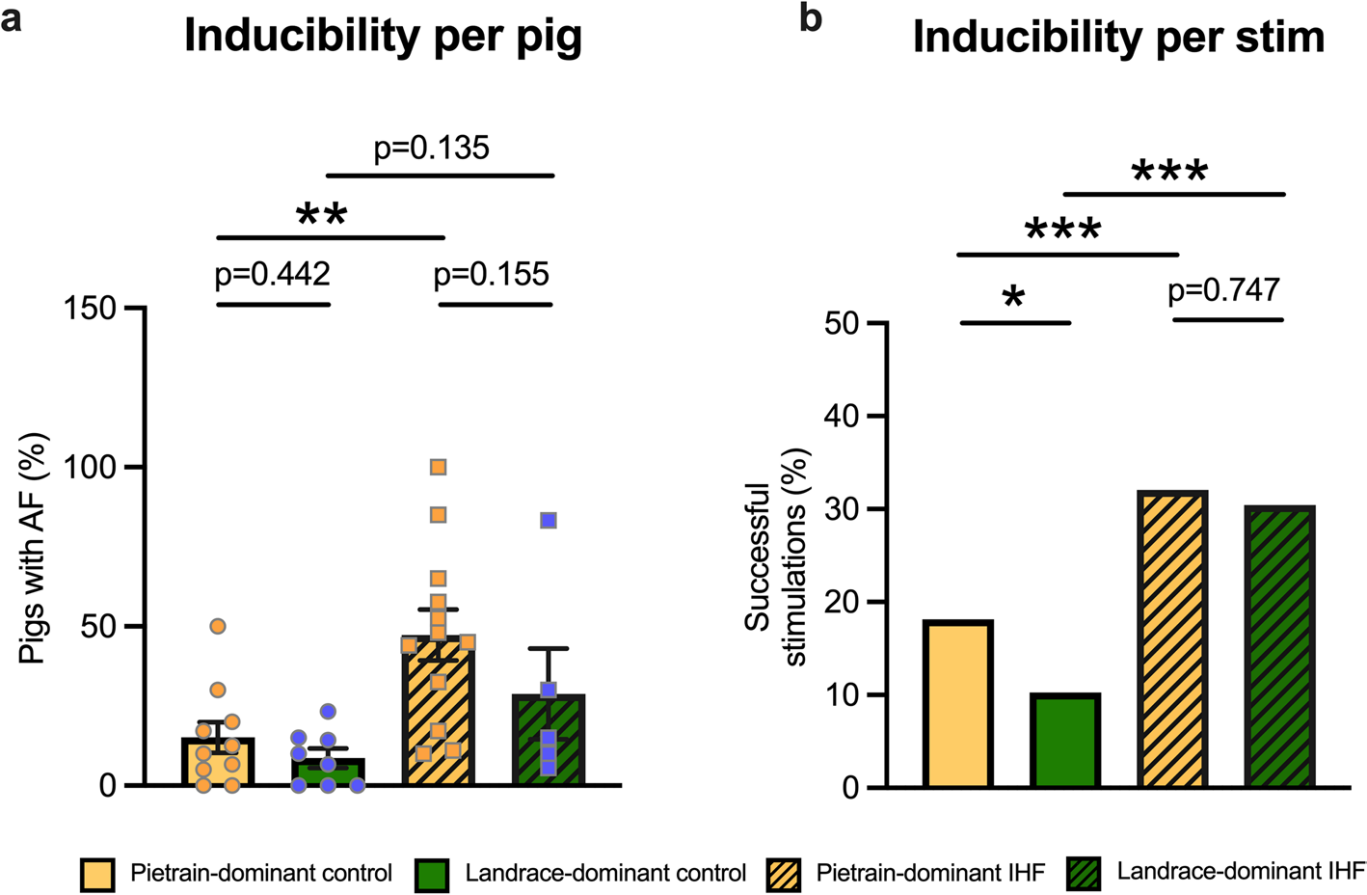
Atrial fibrillation (AF) inducibility. **a** Percentage of pigs with AF episodes longer than 10 s; **b** percentage of successful burst stimulations leading to AF episodes longer than 10 s. Bar graphs represent mean ± SEM, and each circle represents individual pig data. Fisher’s exact test (**a** and **b**). *p < 0.05, **p < 0.01, ***p < 0.001

IHF significantly increased AF susceptibility in Pietrain-dominant pigs, while a non-significant but upward trend was observed in Landrace-dominant pigs, with 47% of Pietrain-dominant pigs and 29% of Landrace-dominant pigs developing AF (*p* < 0.05 for Pietrain-dominant pigs compared to breed-matched controls; Fig. 4a). Correspondingly, a greater proportion of burst stimulations resulted in AF in IHF animals—32% in Pietrain-dominant and 30% in Landrace-dominant pigs—showing a significant increase relative to their respective controls (*p* < 0.001 for Pietrain-dominant and Landrace-dominant; Fig. 4b). Notably, a significant difference in the number of burst stimulations required to induce AF was observed between Pietrain-dominant and Landrace-dominant control pigs (Fig. 4b).

### Electrophysiology studies

Relative sinus node recovery time (SNRT/BCL) and AV Wenckebach cycle length did not significantly differ across groups (Table 2). However, Landrace-dominant IHF pigs showed a significantly shorter AERP at cycle lengths of 450 ms, and 350 ms (Table 2). In contrast, AERP in Pietrain-dominant IHF pigs remained unchanged compared to their controls at any pacing cycle length (Table 2). Moreover, Pietrain-dominant IHF pigs showed a significantly prolonged AVERP at cycle lengths of 450 ms, and 400 ms compared to the controls, while AVERP at cycle lengths of 500 ms and 400 ms in Landrace-dominant IHF pigs was significantly shorter than in Pietrain-dominant IHF pigs (Table 2).

**Table 2:** Summary of electrophysiological properties

	Pietrain-dominant control (*n* = 10)	Pietrain-dominant IHF (*n* = 15)	Landrace-dominant control (*n* = 8)	Landrace-dominant IHF (*n* = 7)
Sinus node recovery time (SNRT)				
SNRT500/BCL (%)	134.7 ± 26.4	158.0 ± 16.3	125.5	123.0 ± 3.3
SNRT450/BCL (%)	141.5 ± 24.9	174.2 ± 18.3	133.6	126.6 ± 5.5
SNRT400/BCL (%)	149.9 ± 31.2	180.2 ± 24.6	148.5	122.2 ± 8.8
Atrial effective refractory period (AERP)				
AERP500 (ms)	227.1 ± 27.5	210.0 ± 15.8	230.0 ± 15.6	186.0 ± 10.3
AERP450 (ms)	213.3 ± 20.5	230.0 ± 11.8	235.0 ± 12.1	192.0 ± 7.3*
AERP400 (ms)	207.8 ± 17.9	207.3 ± 11.9	217.5 ± 11.9	183.3 ± 10.2
AERP350 (ms)	202.2 ± 17.4	192.7 ± 10.9	213.8 ± 11.0	178.3 ± 4.8*
AERP300 (ms)	185.0 ± 15.4	193.1 ± 12.4	205.0 ± 13.4	168.3 ± 11.1
AERP250 (ms)	188.6 ± 19.2	180.0 ± 14.2	186.0 ± 9.8	172.0 ± 14.6
AV Wenckebach cycle length (ms)	212.5 ± 7.5	247.1 ± 12.5	230 ± 10.0	237.5 ± 13.2
AV node effective refractory period (AVERP)				
AVERP500 (ms)	185.0 ± 49.5	271.4 ± 11.2	260.0 ± 10.0	210.0 ± 10.0^&^
AVERP450 (ms)	190.0 ± 25.2	265.7 ± 13.1**	243.3 ± 18.6	226.7 ± 20.3
AVERP400 (ms)	180.0 ± 23.1	251.4 ± 11.0**	240.0 ± 25.2	200.0 ± 11.6^&^
AVERP350 (ms)	175.0 ± 28.6	220.0 ± 13.6	226.7 ± 21.9	200.0 ± 10.0
AVERP300 (ms)	167.5 ± 15.7	184.0 ± 16.0	206.7 ± 3.3	196.7 ± 14.5
AVERP250 (ms)	166.7 ± 32.7	175.0 ± 15.6	190.0	225.0 ± 15.0

Statistical comparisons were performed using the Mann–Whitney *U* test

^*^p < 0.05, **p < 0.01 IHF vs. control pigs. ^&^*p* < 0.05, Landrace-dominant vs. Pietrain-dominant IHF pigs

### Expression profile of fibrosis-related genes

**Fig. 5:**
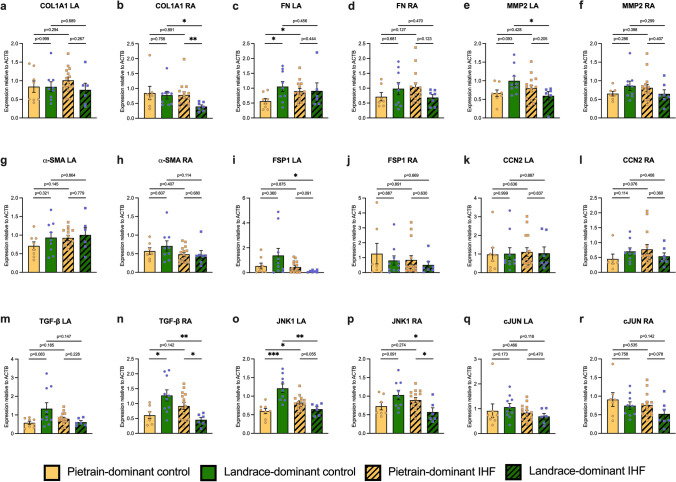
Gene expression analysis of fibrosis-related genes in the left atria (LA) and right atria (RA). **a** Expression of *COL1A1* in LA; **b** expression of *COL1A1* in RA; **c** expression of *FN* in LA; **d** expression of *FN* in RA; **e** expression of *MMP2* in LA; **f** expression of *MMP2* in RA; **g** expression of *ACTA2* in LA; **h** expression of *ACTA2* in RA; **i** expression of *FSP1* in LA; **j** expression of *FSP1* in RA; **k** expression of *CCN2* in LA; **l** expression of *CCN2* in RA; **m** expression of *TGFB* in LA; **n** expression of *TGFB* in RA; **o** expression of *JNK1* in LA; **p** expression of *JNK1* in RA; **q** expression of *cJUN* in LA; **r** expression of *cJUN* in RA. Bar graphs represent mean ± SEM, and each circle represents individual pig data. Mann–Whitney *U* test. *p < 0.05, **p < 0.01, ***p < 0.001

To further investigate the underlying mechanisms driving the distinct differences in structural remodeling observed between Pietrain-dominant and Landrace-dominant pigs in response to IHF, we assessed the expression of key fibrosis-related genes in both the left and right atria (Fig. 5 and Table 3). In Pietrain-dominant IHF pigs, gene expression changes were mainly observed in the left atrium, where fibronectin (*FN*) and *JNK1* were significantly upregulated compared to Pietrain-dominant control pigs (Fig. 5c and o), indicating enhanced ECM deposition and activation of profibrotic signaling. In the right atrium, genes related to ECM remodeling (*FN* and *MMP2*) and profibrotic signaling (*CCN2*, *TGFB*, and *JNK1*) showed a clear upward trend, although these changes did not reach statistical significance (Fig. 5). In contrast, Landrace-dominant pigs with IHF demonstrated a distinctly different expression profile. In the left atrium, the expression of *MMP2*, *FSP1*, *TGFB*, and *JNK1* was significantly reduced compared to Landrace-dominant control pigs (Fig. 5e, i, m, and o), indicating suppressed ECM remodeling and profibrotic signaling. Similarly, in the right atrium, *COL1A1*, *TGFB*, and *JNK1* were significantly downregulated compared to Landrace-dominant control pigs (Fig. 5b, n, and p). Direct comparisons between groups further highlighted these differences. *MMP2* and *JNK1* expression levels in the left atrium, as well as *COL1A1*, *TGFB*, and *JNK1* in the right atrium, were markedly higher in Pietrain-dominant IHF pigs compared to those in Landrace-dominant IHF pigs (Fig. 5b, e, n–p). While not all differences reached statistical significance, the overall gene expression pattern indicates a trend toward more profibrotic signaling in Pietrain-dominant IHF pigs (Table 3).

**Table 3:**
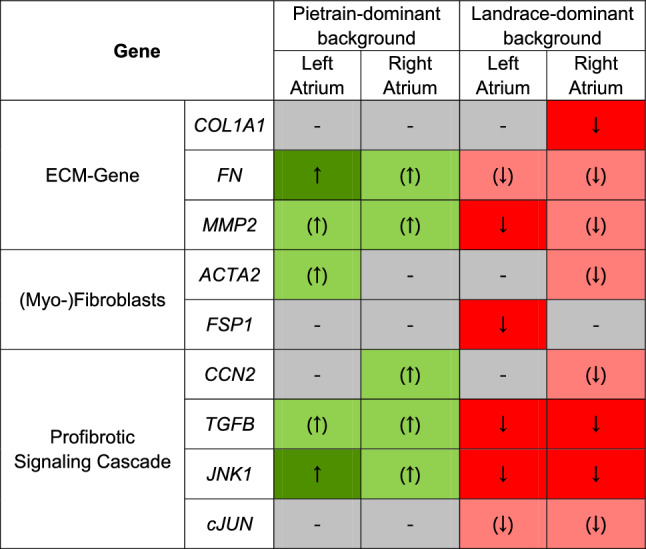
Summary of gene expression analysis

Significant upregulation between IHF and control pigs is indicated by an arrow and green shading

A trend towards upregulation is indicated by an arrow in brackets and light green shading. Significant downregulation is indicated by an arrow and red shading. A trend toward downregulation is indicated by an arrow in brackets and light red shading

## Discussion

The key finding of our study is the identification of distinct atrial remodeling patterns associated with specific genetic backgrounds. Given the retrospective nature of the genetic stratification and the absence of prospective genotype-based group allocation/power calculation, this study should be regarded as hypothesis-generating and exploratory in nature. Although the ischemic trigger and the resulting IHF phenotype were comparable across all animals, the extent and nature of atrial remodeling differed markedly. Genetic background screening revealed a genetically mixed cohort predominantly composed of pigs with Pietrain-dominant and German Landrace-dominant backgrounds, respectively. Importantly, this genetic stratification was performed retrospectively in an already completed experimental cohort and was not used for prospective group allocation or power calculations. Accordingly, the present analysis should be regarded as exploratory and hypothesis-generating. While this design precludes causal conclusions, it enables the identification of biologically relevant patterns that may otherwise remain unrecognized in genetically heterogeneous large-animal studies. Hemodynamic parameters did not differ significantly between breeds in either control or IHF groups. All animals underwent identical surgical procedures, housing, and peri-procedural management, and no systematic differences in infarct location, global cardiac function, or major hemodynamic indices were observed between genetic groups, reducing the likelihood that procedural or environmental confounders account for the observed differences. Following IHF induction, both breeds exhibited similarly increased AF inducibility per stimulation. However, only Pietrain-dominant pigs demonstrated a statistically significant increase in AF inducibility per animal compared to controls, whereas this increase did not reach significance in the Landrace-dominant group. While the percentage of successful burst stimulations inducing AF was comparable between groups, the proportion of animals developing AF differed, further supporting the hypothesis that genetic background contributes to individual variability in AF susceptibility following ischemic injury. These two parameters represent complementary but distinct measures of AF inducibility: animal-level analysis reflects whether an individual is susceptible to AF induction at least once, whereas stimulation-level analysis provides an integrated measure of overall vulnerability across repeated pacing challenges. Given the limited subgroup size—particularly in Landrace-dominant pigs—statistical power differs between these analyses, and effects are more readily detected at the stimulation level due to the higher number of observations. Accordingly, both readouts are presented to provide a more balanced assessment of AF inducibility and to avoid overinterpretation based on a single metric. Given the experimental design, the present data primarily reflect vulnerability to AF initiation rather than the complexity or stability of the underlying arrhythmogenic substrate required for sustained AF maintenance.

Strikingly, Pietrain-dominant pigs developed pronounced atrial fibrosis after IHF, whereas fibrosis was not increased in Landrace-dominant pigs. In addition, Landrace-dominant pigs displayed low heterogeneity of left atrial fibrosis, which did not correlate with AF episodes. This suggests that structural remodeling may represent a major AF substrate in Pietrain-dominant pigs, whereas in Landrace-dominant pigs, ischemic heart failure is associated with changes in atrial refractoriness consistent with electrical remodeling. However, these electrophysiological alterations should be interpreted as indirect in vivo indicators rather than definitive evidence of a dominant electrical substrate.

In clinical practice, significant variations in the incidence, prevalence, phenotype, and response to various triggers of AF have been observed across different ethnic groups [36]. While numerous potential factors have been proposed to explain this variability, a definitive explanation remains elusive. AF is strongly associated with risk factors, such as coronary artery disease or ischemic heart failure. Notably, despite the higher prevalence of these conditions in South Asian populations, the incidence of AF remains lower compared to Caucasian populations [36], supporting the concept that genetic factors modulate AF susceptibility beyond traditional risk factors.

While extensive murine studies have demonstrated strain-dependent differences in cardiac remodeling and AF-related phenotypes [57], translation to human disease is limited by fundamental interspecies differences in cardiac structure and electrophysiology [46]. Large-animal models are, therefore, essential to bridge this gap. The porcine heart closely resembles the human heart in size, anatomy, and electrophysiological properties [10], making it particularly suitable for studying ischemia-induced AF. In this study, we employed a porcine model of IHF to investigate the genetic contribution to proarrhythmic remodeling in AF. Myocardial ischemia is a clinically significant trigger of AF, with patients post MI developing AF in up to 21% of cases [5]. Furthermore, our ischemia protocol mimics the clinical scenario in which most patients with acute myocardial infarction in industrialized countries undergo revascularization ideally within a 90-min window following coronary occlusion. Even within this time frame, ischemic injury can lead to the development of IHF [33]. The model successfully reproduced this clinical scenario, resulting in a robust IHF phenotype characterized by reduced ejection fraction and altered hemodynamics in both atria and ventricles, coupled with an increased susceptibility to AF.

The genetic contribution to cardiac structural remodeling under both baseline and pathological conditions is well established [48]. Murine studies have demonstrated pronounced strain-dependent differences in hypertrophy and fibrosis following pressure overload or neurohumoral stress, including after transverse aortic constriction and chronic angiotensin II administration [2, 9]. Using the Hybrid Mouse Diversity Panel, Rau, Wang, and colleagues further showed substantial inter-strain variability in ventricular fibrosis and function in isoproterenol-induced heart failure [43, 59], underscoring the role of genetic background in modulating cardiac remodeling. In line with these findings, porcine studies have also suggested that genetic background influences ischemic outcomes, even in the absence of overt macroscopic differences following myocardial ischemia [50]. Building on these findings, our data demonstrate that genetic background also critically affects atrial remodeling trajectories following ischemic heart failure. Importantly, our results extend previous porcine ischemia/reperfusion studies by showing that genetic background not only influences infarct tolerance and vascular function but also shapes atrial remodeling and arrhythmogenic substrates. Prior work in Ossabaw minipigs identified a genetically determined non-responder phenotype to ischemic preconditioning, associated with impaired activation of mitochondrial and STAT3-dependent cardioprotective signaling [27, 29]. Complementary studies demonstrated genetically fixed alterations in vasomotor function that may exacerbate microvascular dysfunction during reperfusion [17, 28]. In this context, our findings show that comparable ischemic injury elicits divergent atrial phenotypes depending on genetic background, resulting in fibrosis-dominated remodeling in Pietrain-dominant pigs.

In addition to structural remodeling, we observed marked differences in atrial electrophysiology between the two breeds. While Landrace-dominant pigs exhibited a significant reduction in AERP, no such change was observed in Pietrain-dominant pigs, suggesting a predominant role of electrical remodeling in the former. Several alternative and potentially complementary mechanisms may contribute to the observed electrophysiological phenotype in Landrace-dominant pigs. Autonomic nervous system modulation, which is known to influence atrial refractoriness and AF susceptibility [4, 54], was not assessed in the present study and may differ between genetic backgrounds. In addition, atrial stretch related to elevated filling pressures in the setting of ischemic heart failure could alter electrophysiological properties independently of overt fibrosis [19, 45]. Finally, subtle structural changes at the microstructural level—such as diffuse interstitial remodeling, alterations in myocyte coupling, or extracellular matrix changes below the resolution of standard histological quantification—may contribute to arrhythmogenicity without manifesting as measurable increases in interstitial fibrosis [16, 31]. The influence of genetic background on electrophysiological properties has been extensively characterized in mice, where significant inter-strain differences in AERP have been reported in Langendorff-perfused hearts from C57BL/6, FVB/N, MF1, 129/SV, and Swiss Agouti strains [37]. These disparities are thought to arise from variations in intracellular calcium handling and ion channel expression [37]. For example, Balb/c mice exhibit heightened sarcoplasmic reticulum calcium leak compared to other strains [49], while sodium current amplitudes in neonatal cardiomyocytes differ among C57BL/6, FVB/N, and 129/Sv strains [32]. Additionally, *Kcnc4* expression has been shown to vary across mouse strains, further influencing atrial electrophysiology [25]. Our in vivo findings in pigs align with these ex vivo mouse studies, reinforcing the notion that the genetic background modulates atrial electrophysiological properties and, consequently, AF vulnerability.

In progressive heart failure, activation of profibrotic signaling pathways leads to interstitial fibrosis in both ischemic and non-ischemic regions, including the atria, thereby promoting conduction disturbances and reentry [7, 22]. To explore molecular correlates of the observed breed-specific differences in atrial fibrosis, we analyzed the expression of fibrosis-associated genes. Fibronectin (FN), a key regulator of extracellular matrix organization [51], was significantly upregulated in Pietrain-dominant IHF pigs but not in Landrace-dominant pigs, whereas MMP2 expression was reduced in the left atria of Landrace-dominant pigs. The balance between extracellular matrix turnover and profibrotic signaling—particularly involving TGF-β and downstream MAPK pathways—is a critical determinant of cardiac fibrosis [18, 24]. Consistent with this, TGF-β expression was reduced in Landrace-dominant pigs, while JNK1 showed opposing regulation between genetic backgrounds. These transcriptional patterns are in line with the previous reports in human ischemic cardiomyopathy and experimental heart failure models [8, 23, 55, 61], and suggest divergent molecular responses to ischemic injury across genetic backgrounds, although not all changes reached statistical significance. The differential expression of these genes in Pietrain- and Landrace-dominant pigs identifies transcriptional signatures associated with the breed-specific differences in atrial fibrosis observed in our study. Nevertheless, given the retrospective nature of the genetic stratification, these molecular and phenotypic associations should not be interpreted as definitive evidence of causality but rather as converging signals supporting a modulatory role of genetic background.

Despite these findings, our study has several important limitations that should be carefully considered. Most notably, the genetic background analysis was performed retrospectively in an already completed experimental cohort, and animals were not prospectively stratified or powered based on genotype. This design inherently increases the risk of post hoc subgrouping and limits causal inference. While all animals underwent identical experimental procedures, housing, and peri-procedural management and developed a comparable ischemic heart failure phenotype, we cannot fully exclude the possibility that subtle, unrecognized confounders may correlate with genetic background. Consequently, the present findings should be interpreted as hypothesis-generating rather than definitive, and prospective studies with predefined genetic stratification will be required to confirm and extend these observations. Second, mechanistic interrogation of electrical remodeling was limited to in vivo electrophysiological measurements. Cellular electrophysiology, ion channel profiling, calcium handling analyses, and high-resolution atrial mapping were beyond the scope of this retrospective study. As such, electrical remodeling in Landrace-dominant pigs should be interpreted as suggested by functional electrophysiological parameters rather than as a definitively established substrate. Third, while the overall sample size is robust for a large-animal model, subgroup sizes may still limit the statistical power to draw definitive conclusions, particularly in genetically stratified analyses. Fourth, although our gene expression profiling revealed key molecular alterations associated with AF susceptibility, we were unable to pinpoint a single gene with clear biomarker or therapeutic potential, and protein-level validation, direct assessment of signaling pathway activity (e.g., TGF-β signaling), and functional assays were not performed and, therefore, limit mechanistic interpretation of the gene expression data. Fifth, although the overall cohort size is substantial for a large-animal study, genetic stratification resulted in smaller subgroup sizes, which limits statistical power and increases the risk of type II error. This is particularly relevant for molecular and electrophysiological analyses involving multiple endpoints. Accordingly, the absence of statistically significant differences should be interpreted cautiously. Future studies using larger, prospectively powered cohorts and integrative omics approaches are warranted to uncover novel, clinically translatable targets for atrial fibrillation in the setting of ischemic heart failure.

In conclusion, our findings demonstrate that while IHF increases AF susceptibility in both Pietrain- and Landrace-dominant pigs, distinct atrial remodeling patterns emerge. Pietrain-dominant IHF pigs exhibit significant atrial fibrosis, whereas Landrace-dominant IHF pigs predominantly undergo electrical remodeling, as reflected by a shortened AERP. These findings provide evidence that genetic background is associated with differences in the atrial remodeling response to ischemia and the resulting AF substrate. Future investigations should focus on elucidating the precise molecular and electrophysiological pathways driving these breed-specific differences. A deeper understanding of the genetic modulation of proarrhythmic remodeling will facilitate personalized AF risk prediction and enable the development of targeted therapeutic strategies. Moreover, our findings underscore the importance of considering genetic background in the design and interpretation of preclinical studies using porcine models of AF.
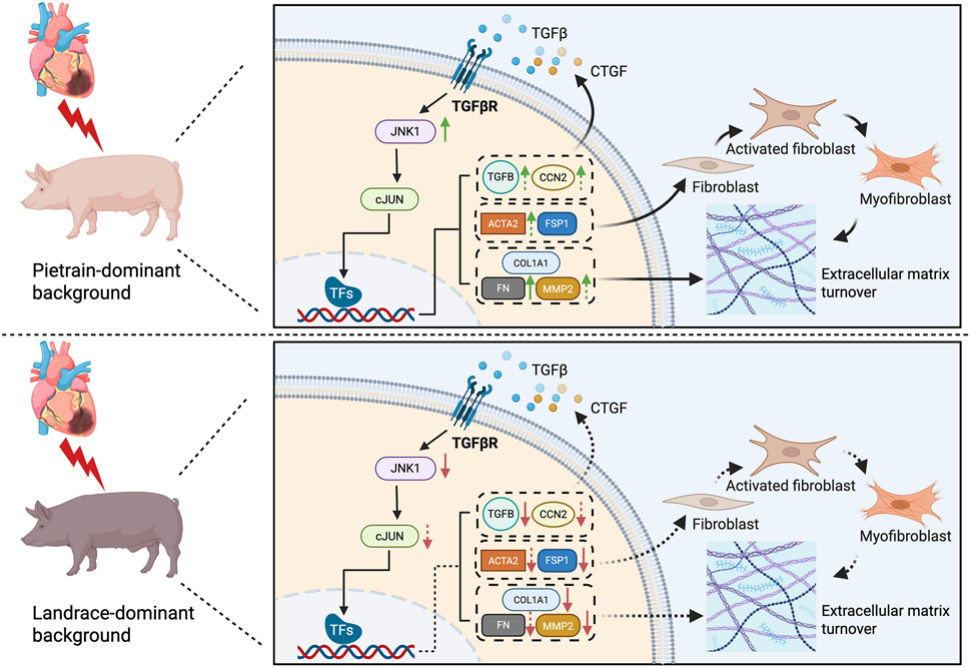


## Data Availability

The data that support the findings of this study are available from the corresponding author upon request.
